# Profound tumor-specific Th2 bias in patients with malignant glioma

**DOI:** 10.1186/1471-2407-12-561

**Published:** 2012-11-27

**Authors:** Shinji Shimato, Lisa M Maier, Richard Maier, Jeffrey N Bruce, Richard CE Anderson, David E Anderson

**Affiliations:** 1 Department of Neurosurgery, Gabriele Bartoli Brain Tumor Research Laboratory, Columbia University, New York City, NY, USA; 2 Erinyes Biotechnologies, Inc, Boston, MA, USA; 3 Department of Ophthalmology, Medical University of Graz, Graz, Austria

## Abstract

**Background:**

Vaccination against tumor-associated antigens is one promising approach to immunotherapy against malignant gliomas. While previous vaccine efforts have focused exclusively on HLA class I-restricted peptides, class II-restricted peptides are necessary to induce CD4^+^ helper T cells and sustain effective anti-tumor immunity. In this report we investigated the ability of five candidate peptide epitopes derived from glioma-associated antigens MAGE and IL-13 receptor α2 to detect and characterize CD4^+^ helper T cell responses in the peripheral blood of patients with malignant gliomas.

**Methods:**

Primary T cell responses were determined by stimulating freshly isolated PBMCs from patients with primary glioblastoma (GBM) (n = 8), recurrent GBM (n = 5), meningioma (n = 7), and healthy controls (n = 6) with each candidate peptide, as well as anti-CD3 monoclonal antibody (mAb) and an immunodominant peptide epitope derived from myelin basic protein (MBP) serving as positive and negative controls, respectively. ELISA was used to measure IFN-γ and IL-5 levels, and the ratio of IFN-γ/IL-5 was used to determine whether the response had a predominant Th1 or Th2 bias.

**Results:**

We demonstrate that novel HLA Class-II restricted MAGE-A3 and IL-13Rα2 peptides can detect T cell responses in patients with GBMs as well as in healthy subjects. Stimulation with a variety of peptide antigens over-expressed by gliomas is associated with a profound reduction in the IFN-γ/IL-5 ratio in GBM patients relative to healthy subjects. This bias is more pronounced in patients with recurrent GBMs.

**Conclusions:**

Therapeutic vaccine strategies to shift tumor antigen-specific T cell response to a more immunostimulatory Th1 bias may be needed for immunotherapeutic trials to be more successful clinically.

## Background

Passionate debate for over 100 years [1] has surrounded the concept that host immunity can protect against tumor development without external therapeutic intervention. This concept – cancer immunosurveillance –named and further developed in the late 1950s by Burnet and Thomas [2,3] was more recently refined by Schreiber and colleagues to reflect the additional influence host immunity has in shaping the immunological phenotype of tumor cells, a concept called cancer immunoediting [4]. A large body of animal and human data now provide convincing support for the ability of host immunity to suppress tumor development [5].

Nevertheless, cancer vaccines have thus far overwhelmingly failed to induce objective clinical responses [6]. There are many potential explanations for the lack of efficacious therapeutic cancer vaccines, including the challenges of inducing potent immunity given the poor inherent immunogenicity of most tumor-associated antigens, the presence of regulatory T cell populations, and the immunosuppressive tumor environments in which tumor-specific T cells are needed to exert their function [7,8]. Additionally, in spite of studies demonstrating the importance of T helper cells in sustaining effective CTL responses [9,10], early cancer vaccines have been overly focused on assessing and improving tumor-specific CTL responses with comparatively little attention paid to T helper cell responses [11,12]. Peptide-based cancer vaccines in particular have overwhelmingly relied on only one or a few HLA class I-restricted cytotoxic T lymphocyte (CTL) epitopes typically without inclusion of any HLA class II-restricted T helper cell epitopes [13,14].

In this study, we focused on five candidate peptide epitopes derived from MAGE family and interleukin-13 receptor α2 (IL-13Rα2) antigens that were designed for recognition by class II-restricted T helper cells. We tested the ability of our selected candidate peptides to induce T cell responses in peripheral blood from patients with GBMs by measuring IFN-γ and IL-5 as type 1 and 2 T helper cell cytokines, respectively. Our results demonstrate that our candidate peptides detected CD4^+^ T helper cell responses in patients with GBMs as well as healthy subjects. While helper T cell responses in healthy subjects had a clear Th1 bias, T cell responses in patients with GBMs demonstrated a markedly Th2-skewed response, which was even more pronounced in patients with recurrent GBMs. These findings suggest that future vaccination strategies that stimulate both class I and class II restricted T cells, as well as reverse the profound Th2 skewing, may be needed to have significant clinical efficacy in patients with GBMs.

## Methods

### Peptides

All peptides were synthesized using standard FMOC chemistry to 95% purity (New England Peptide Company). All peptides were at least 15 amino acids in length. The following peptides were used for stimulation of PBMCs: MAGE-A3_112-127_ (KVDELAHFLLRKYRAK); MAGE-A3_121-136_ (LRKYRAKELVTKAEML); MAGE-A3_143-160_ (WQYFFPVIFSKASSSLQL); IL13Rα2_341-355_ (LLRFWLPFGFILILV); IL-13Rα2_351-365_ (ILILVIFVTGLLLRK); MBP_85-99_ (ENPVVHFFKNIVTPR). HLA class II alleles predicted to bind the peptides were determined using ProPred HLA class II binding algorithm [15], summarized in Table 1. All peptides were predicted to be very promiscuous, binding to multiple (up to 9 in several cases) alleles. The validity of the predictions is supported by experimental data for the MBP_85-99_ peptide, which has been shown to bind to both the DRB1*04 and *15 alleles as predicted [16].

**Table 1:** Predicted HLA Class II Binding Alleles for the peptides used in this study

**Epitope**	**Predicted HLA Class II Binding Alleles**
MAGE-A3_112-127_	DRB1*04, *08, *11, *13, DRB5*01
MAGE-A3_121-136_	DRB1*04, *07, *08, *11, *13, *15, DRB5*01
MAGE-A3_143-160_	DRB1*01, *03, *04, *07, *08, *11, *13, *15, DRB5*01
IL-13Ra2_341-355_	DRB1*01, *03, *04, *07, *08, *11, *13, *15, DRB5*01
IL-13Ra2_351-365_	DRB1*01, *03, *04, *07, *08, *11, *13, *15, DRB5*01
MBP_85-99_	DRB1*01, *03, *04, *08, *11, *13, *15

Putative HLA-DR binding was determined using the ProPred MHC Class-II binding peptide prediction server, and emphasizes the promiscuous binding of the MAGE-A3 and IL-13Rα2 peptides used in this study.

### Isolation of PBMCs

Twenty-five mL of blood from patients or healthy subjects was obtained under an IRB-approved protocol. All samples of blood from patients were taken at the time of surgery. Ages of the patients ranged from 48 to 76 among patients with primary GBMs (median age 55), from 41 to 69 among patients with recurrent GBMs (median age 52), and from 40 to 73 among patients with meningiomas (median age 62). A greater number of men had primary or recurrent GBMs (6 of 8 patients and 5 of 6 patients, respectively), while more women than men had meningiomas (5 of 8 patients). Primary and recurrent GBMs were located in temporal, parietal, and frontal lobes with comparable frequencies. All tumor-bearing patients received similar doses of steroids and anti-epileptic medications at the time of tumor debulking surgery prior to obtaining peripheral blood for these studies. T cell responses in patients with meningiomas controlled for influences of steroids on antigen responsiveness and cytokine balance. Tumor tissue was independently confirmed in all cases by formal pathological analysis. PBMCs were purified from heparinized blood by density gradient centrifugation using Ficoll-Hypaque (GE Healthcare Biosciences), and cells were then washed with PBS and viable cells quantified by trypan-blue staining.

### PBMC culture with peptides

Freshly isolated PBMCs were plated at 2 × 10^5^ cells/well in 200 μl of serum-free X-VIVO15 (X15) media (Lonza) in 96-well round-bottom cell culture plates. Candidate peptides, in addition to a negative control peptide derived from MBP were added at a concentration of 10 μg/mL and anti-CD3 mAb was added at a concentration of 1 μg/mL. Six T cell cultures were established for each condition in each subject, and 100 IU/ml of IL-2 was added on the following day. Plates were incubated at 37°C and 5% CO_2_ for 14 days, with media changed as needed, and the supernatant was harvested to evaluate T cell responses (cytokines) induced by each condition using ELISA. In a limited number (n = 3) of patients, we assessed cytokine production after both 7 and 14 days. Tumor-specific responses were apparent at day 7, and the frequency of positive responses did not change significantly at day 14, but the cytokine values did increase significantly (data not shown). An IFN-ELISPOT assay was performed as previously described [17].

### Cytokine measurement

To detect T helper cell responses directed against the candidate peptides, IFN-γ and IL-5 were measured by ELISA using commercially available kits supplied by BD bioscience. IFN-γ was used as a prototypic Th1 cytokine and IL-5 was chosen as a prototypic cytokine released by Th2 cells because unlike IL-4 there would be no potential consumption by antigen-specific T cells in our culture conditions [18]. The Th2-associated transcription factor GATA-3 directly binds and regulates both the IL-4 and IL-5 gene promoters [19] and a positive correlation has been reported among GATA-3, IL-4, and IL-5 gene expression during human T cell differentiation [20], providing further support for analysis of IL-5 as a representative Th2 cytokine. Initial experiments also examined the secretion of IL-10 in response to peptide stimulation, which was not detected. Flat-bottom microtiter plates (Immulon) were coated with primary antibody (IFN-γ or IL-5) diluted 1:1000 in NaHCO3 and incubated overnight at 4°C. Coating solution was then removed, plates blocked with PBS + 10%FBS at 25°C for 2 hours, rinsed 3 times with diluted wash buffer (dH20, Tween 20, PBS 20X), and standards were then added in duplicate at 0, 62.5, 125, 250, 500, 1000, 2000, and 4000 pg/mL (diluted in X15 media). Supernatants (50 μl/well) from T cell assays were then added to wells. Plates were incubated for 2 hours at 25°C and subsequently rinsed 3 times. Wells were then coated with a secondary biotinylated antibody diluted 1:1000 in PBS + 1%FBS and incubated for 1 hour at 25°C. Plates were again rinsed 3 times and avidin-peroxidase diluted 1:1000 in PBS+10%FBS was added and incubated for another 1hour. After rinsing 6 times, TMB (tetramethylbenzidine) (BD biosciences) was added to wells, which were allowed to develop. The reaction was stopped by adding 50 μL of sulfuric acid and absorbance was measured at 455 nm by an ELISA plate reader (BIO-RADR). A standard curve was generated by plotting absorbance against each reference standard, and sample concentrations were extrapolated from this curve. Appropriate statistical tests and analyses based on our data were determined using Prism 5.0 (GraphPad software).

## Results

### Global T cell responses

**Figure 1:**
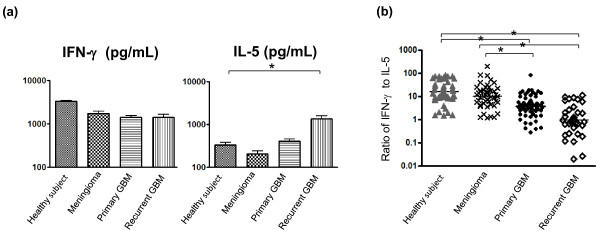
**Global T cell cytokine profiles among patients with CNS tumors and healthy controls.(a)** The geometric mean values and standard deviation of IFN-γ and IL-5 levels from all T cell cultures generated with anti-CD3 mAb among the four groups examined are presented. Modest decreases in the amount of secreted IFN-γ are seen among all patients with CNS tumors when compared to healthy subjects, while a significant elevation of IL-5 levels is seen only in recurrent GBM patients. **(b)** The ratios of IFN-γ to IL-5 for all primary T cell responses are shown for each cohort. There was no difference in this ratio comparing patients with meningiomas to healthy subjects, but patients with primary and recurrent GBM patients exhibited significantly lower ratios compared to both healthy subjects and meningioma patients.

Six primary T cell cultures were established from each patient against all stimuli. Anti-CD3 mAb was used to stimulate and expand T cells to confirm T cell viability and to examine global, nonspecific T cell cytokine responses among the different cohorts. Relative to healthy subjects, anti-CD3 mAb-induced IFN-γ levels in patients with GBMs (primary and recurrent) and meningiomas were modestly lower (Figure 1a). More strikingly, anti-CD3 mAb stimulation uniquely induced secretion of high amounts of IL-5 from patients with recurrent GBMs (P < 0.0001). A recent clinical trial examined the IFN-γ/IL-5 ratio after polyclonal stimulation of PBMCs in patients with metastatic melanoma treated with immunomodulators given to restore the Th1/Th2 balance [21], and we performed a similar analysis of our data (Figure 1b). The IFN-γ/IL-5 ratios in both primary GBM patients (geometric mean 3.7) and recurrent GBMs (geometric mean 0.9) were significantly lower than those in healthy subjects (geometric mean 16.0) and meningioma patients (geometric mean 10.0) (p<0.001). This antigen-nonspecific bias towards a Th2 response in patients with primary and recurrent GBMs is consistent with past reports [22-25]. There was no significant difference in the global IFN-γ/IL-5 ratio between healthy subjects and meningioma patients, indicating that neither treatment with steroids or antiepileptic medications nor the simple presence of a CNS tumor were responsible for the deviation in global T cell responses.

### Responses to HLA Class II-restricted peptide stimulation

Both glial cells and melanocytes derive from neural ectoderm [26] and several studies have demonstrated that melanoma-associated tumor antigens are also expressed by gliomas, including MAGE-A3 [27-29]. Like MAGE-A3, IL-13Rα2 is a cancer testes antigen that is over-expressed in gliomas [30,31]. In trying to identify novel glioma-associated HLA class II-restricted T helper cell epitopes, we hypothesized that epitopes identified in patients with melanoma may similarly be expressed by patients with gliomas. Two such epitopes with homology to MAGE-A3 were identified [32-34], which we modified to incorporate adjacent HLA class I-restricted CTL epitopes [35-38] as well as several amino acid substitutions. The amino acid substitutions altered the hydrophobicity of the peptides but not their charge (Ala to Asp, Leu to Arg) and potentially their secondary structure (Pro to Leu). Similarly, two overlapping 15mer IL-13Rα2 epitopes were identified, one of which was modified to incorporate a CTL epitope [39]. The five epitopes used in this study in relation to previously described epitopes are depicted in Table 2.

**Table 2:** Candidate glioma-associated T helper cell epitopes

	
MAGE-A3_112-160_	**KVAELVHFLLLKYRAREPVTKAEMLGSVVGNW QYFFPVIFSKASSSLQL**
MAGE-A3_112-127_	*KV****D****EL****A****HFLL***R**KYRA**K**
MAGE-A3_121-136_	L**R**KYRA***K****E****L****VTKAEML*
MAGE-A3_143-160_	*WQYFFPVIF*SKASSSLQL
MAGE-A3_121-134_	FLLLKYRAREPVTKAE
MAGE-A3_146-160_	FFPVIFSKASSSLQL
IL-13Rα2_341-365_	**LLRFWLPFGFILILVIFVTQLLLRK**
IL-13Rα2_341-355_	LLRF*WLPFGFILI*LV
IL-13Rα2_351-365_	ILILVIFVTQLLLRK

**Figure 2:**
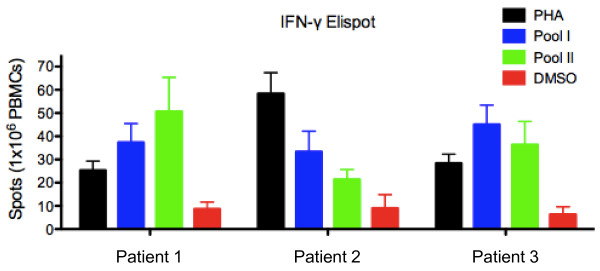
**Memory T cell responses against GBM peptide antigens detected by ELISPOT.** The MAGE-A3 peptides (MAGE-A3_112-127_, MAGE-A3_121-136_, and MAGE-A3_143-160_ were dissolved in DMSO in equimolar amounts (peptide pool I) while the IL13Rα2 peptides IL13Rα2_341-355_ and IL-13Rα2_351-365_ were similarly dissolved together (peptide pool II) and used to stimulate freshly isolated PBMCs from 3 patients with primary GBMs. Significant (p < 0.05) responses to both peptide pools were detected in all patients. Mean + SD are presented.

Measurement of antigen-specific T cell responses in the peripheral blood in humans differs depending on whether responses are high affinity interactions with foreign (viral) epitopes or lower affinity interactions involving recognition of self-antigens. We have previously demonstrated that high frequencies of T cells directed against the self-antigen MBP peptide 85–99 in the peripheral blood of patients with multiple sclerosis (MS) fail to proliferate when stimulated with antigen but readily secrete high levels of cytokine [40]. Given that T cell responses directed against MAGE and IL-13Rα2 antigens also involve T cells with low affinity to these self-antigens, we quantified antigen-specific responses based on cytokine secretion, as recently described in a phase I study of patients with MS [41]. We quantified cytokine production by ELISA, defining a positive T cell response for each patient as the amounts of IFN-γ or IL-5 that were > 50 pg/mL and two standard deviations above the mean cytokine levels secreted after stimulation of cells from that patient with negative control MBP peptide. The mean cut-off for a positive cytokine response based on cytokine induced by stimulation with control MBP peptide was 895 pg/ml (range: 13–1298) and 314 pg/ml (range: 72–852) for IFN-γ and IL-5 among healthy subjects, and was 123 pg/ml (range: 0–286) and 312 pg/ml (range: 59–1347) for IFN-γ and IL-5 among GBM patients. Use of a traditional IFN-γ ELISPOT assay, in which quantification of spots can at times be ambiguous, confirmed that memory T cell responses could be detected with these peptides in patients with primary GBMs, as peptide specific cytokine production could be detected with 48 hours of culture (Figure 2).

**Figure 3:**
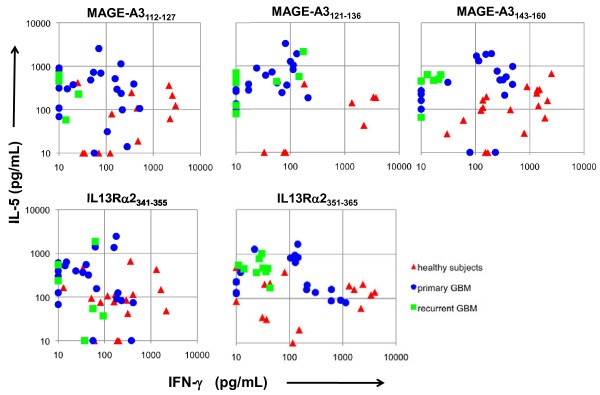
**T cell cytokine profiles to each peptide among each cohort.** Each symbol represents the IFN-γ and IL-5 cytokine levels for a positive T cell response, defined as greater than 50 pg/ml and two standard deviations above the mean cytokine levels secreted after stimulation of cells with negative control MBP peptide for each subject. The mean cut-off for a positive cytokine response based on cytokine induced by stimulation with control MBP peptide was 895 pg/ml (range: 13–1298) and 314 pg/ml (range: 72–852) for IFN-γ and IL-5 among healthy subjects, and was 123 pg/ml (range: 0–286) and 312 pg/ml (range: 59–1347) for IFN-γ and IL-5 among GBM patients.

**Figure 4:**
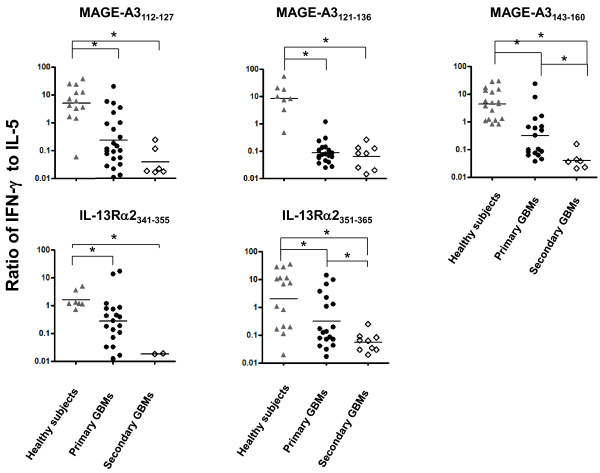
**Th1/2 ratios of T cell responses to each peptide among each cohort.** The ratio of IFN-γ to IL-5 for each primary T cell response presented in Figure 3 is presented. Patients with primary GBMs had significantly lower ratios compared to healthy subjects for every antigen examined. Patients with recurrent GBMs had significantly lower ratios compared to patients with primary GBMs in response to the MAGE-A3_143-160_ and IL-13Rα2_351-365_ epitopes (p<0.05).

T cells responding to all five peptides examined among healthy subjects exhibited a predominant Th1 response (high IFN-γ and low IL-5 secretion) (Figure 3). In marked contrast, the majority of peptide-specific T cell responses among both primary and recurrent GBM patients were Th2 polarized (low IFN-γ and high IL-5 secretion). Frequencies of response to the individual peptides were most prevalent among healthy subjects and patients with primary GBMs, both in terms of the number of subjects responding to a given peptide and the number of positive lines. Responses in one or a few subjects did not dominate among any of the cohorts examined; at least half of the subjects (in some cases all) in each cohort responded to all of the epitopes tested (Table 3). Patients with meningiomas generally had less frequent responses, though strong responses could be detected against both MAGE-A3 and IL-13Rαpeptides. Mean IFN-γ/IL-5 ratios were significantly lower (p<0.05) for patients with primary GMBs (geometric means for MAGE-A3_112-127_, MAGE-A3_121-136_, MAGE-A3_143-160_, IL13Rα2_341-355_, and IL-13Rα2_351-365_ were 0.2, 0.1, 0.3, 0.3, 0.3, respectively) relative to healthy subjects (geometric means were 4.9, 8.1, 4.3, 1.6, 2.0, respectively) in response to all of the epitopes (Figure 4). The Th2 bias was even more profound among patients with recurrent GBMs (geometric means for MAGE-A3_112-127_, MAGE-A3_121-136_, MAGE-A3_143-160_, IL13Rα2_341-355_, and IL-13Ra2_351-365_ were 0.04, 0.06, 0.4, 0.02, 0.05), and was significantly lower than that of patients with primary GBMs for the MAGE-A3_143-160_ and IL-13Rα2_351-365_ epitopes (P<0.05).

**Table 3:** Frequencies of response among subjects to the candidate glioma-associated T helper cell epitopes

	**MAGE-A3**_**112-127**_	**MAGE-A3**_**121-136**_	**MAGE-A3**_**143-160**_	**IL-13Rα2**_**341-355**_	**IL-13Rα2**_**351-365**_
Primary GBM 1		++	++++	+	+++
Primary GBM 2	++	++	+++	++	++
Primary GBM 3	++	++++	+	+++++	++
Primary GBM 4	+	++	+		+++
Primary GBM 5	+++++	++++	+	++	+
Primary GBM 6	++	++	+	++	
Primary GBM 7	+++	++++	++	+++	+++
Primary GBM 8	++++	++	+++++	++++	+++++
Recurrent GBM 1	+	++	+	++	++
Recurrent GBM 2	+				
Recurrent GBM 3	++	+	+		+
Recurrent GBM 4	++	+	+		
Recurrent GBM 5	+	+			++
Meningioma 1		+			
Meningioma 2		+++			
Meningioma 3					+
Meningioma 4			+		
Meningioma 5	+++			++	++
Meningioma 6	+	+	+	+	++++++
Meningioma 7			+		
Healthy Subject 1	+++	+++	+++		+
Healthy Subject 2		+	+++	+	
Healthy Subject 3	++	+++	+		
Healthy Subject 4	++	+	+++++	+	+++++
Healthy Subject 5	+	+	++	++++	++
Healthy Subject 6		+	+	+	++

Cytokine production was quantified by ELISA, defining a positive T cell response for each patient as the amounts of IFN-γ or IL-5 that were > 50 pg/mL and two standard deviations above the mean cytokine levels secreted after stimulation of cells from that patient with negative control MBP peptide. A total of 6 primary T cell responses were measured for each subject against each peptide. (+) symbols indicate the precise number of positive wells among six for each peptide.

## Discussion

Cytokines, while not directly cytolytic to tumor cells, can create strong negative pressure on tumor cell survival. Noteworthy are observations that mice unable to secrete or respond to the cytokine IFN-γ are more susceptible to chemically-induced and spontaneous tumors [42], and that in humans Th1 responses among tumor infiltrating lymphocytes are associated with favorable clinical outcome [43]. Thus, secretion of IFN-a cytokine that defines Th1 cells, is an important effector molecule involved in tumor immunity. By contrast, Th2 cells secrete cytokines such as IL-4, IL-5, and IL-13, and the characteristic cytokines produced by Th1 and Th2 cells are mutually inhibitory for the differentiation and effector functions of the reciprocal T cell phenotype. Accordingly, it is prudent to evaluate the Th1/Th2 balance in order to predict the efficacy of helper T cells directed against a tumor. Several reports using both animal models and human tumor specimens have investigated the Th1/2 cytokine balance in patients with gliomas and found a strong Th2 bias [22-25]. However, none of these investigations characterized the T cell-derived, tumor-specific cytokine balance.

In this study we evaluated T helper cell responses against a panel of putative HLA class II-restricted peptides derived from glioma-associated tumor antigens. To our knowledge this is first study to demonstrate glioma-associated, class II-restricted epitopes and to reveal a profound Th2 bias among glioma-specific T helper cells. Th2-skewed immunity specific to carbioembryonic antigen has similarly been observed in patients with pancreatic cancer [44]. We further observed that the Th2 bias in GBM patients is augmented among patients with recurrent GBMs due to enhanced secretion of the Th2 cytokine IL-5. Suppression of Th1 immunity is similarly observed in colorectal cancer patients at a relatively early stage of the disease while increased Th2 immunity appears during terminal stages of disease [45], suggesting that blunting of Th1 responses occurs prior to, not concomitant with, Th2 deviation of tumor-specific immunity. In contrast to responses detected in patients with malignant GBMs, circulating T helper cells directed against the same epitopes were readily detected among healthy subjects with a protective Th1 bias at comparable frequencies.

## Conclusions

Our findings suggest that both primary and recurrent GBM patients are unlikely to have the capacity to favorably respond to immunization against tumor antigens that involve peptide and subunit vaccines with weak ability to promote Th1 immunity. Indeed, suboptimal vaccination could even enhance the immunosuppressive status of patients, as recently demonstrated when HLA class II-restricted peptide vaccination induced regulatory T cells with potential to exacerbate the immunosuppressive state in the patients [46]. In another recent clinical trial conducted in melanoma patients that involved multiple HLA class II-restricted peptides from MAGE and melanocytic differentiation antigen, vaccine-induced T helper cell responses were induced in a majority of the patients (81%), yet beneficial clinical responses were observed in only two out of 17 patients [47].

Studies have demonstrated that resection of tumor or achievement of disease free status can restore Th1 immunity in patients with malignant diseases such as malignant melanomas and renal cell carcinomas [48,49]. This suggests that successful resection of gliomas may reverse an unfavorable background that promotes Th2 bias in these patients, and may represent an ideal time at which to administer a therapeutic vaccine comprised of the HLA class II-restricted glioma-associated antigens that we have defined. Formulation of our GBM peptide antigens with TLR agonists, in particular the TLR9 agonist CpG, could be used to further reverse the Th2 bias directed against these antigens as well as ameliorate the suppressive activity associated with regulatory T cells directed against the same antigens [50-54]. Moreover, the antigens that we have defined may be applicable to vaccination of patients with melanoma, given that MAGE antigens are frequently over-expressed among melanomas, and functional evidence of T helper cell recognition of antigens shared by melanoma and glioma cells [55].

## Competing interests

DEA is listed as an inventor on a vaccine comprised of the peptides identified in this manuscript.

## Authors’ contributions

DEA designed the experimental approach, SS and LMM were responsible for performing the experiments, SS, RCEA, and DEA prepared the manuscript, and RM and JNB provided helpful discussions and critical review of the manuscript. All authors read and approved the final manuscript.

## Pre-publication history

The pre-publication history for this paper can be accessed here:

http://www.biomedcentral.com/1471-2407/12/561/prepub
